# Anæsthesia, or the Employment of Chloroform and Ether in Surgery, Midwifery, Etc.

**Published:** 1849-07

**Authors:** 


*Anæsthesia, or the employment of Chloroform and Ether in Surgery, Midwifery*, etc. By J. Y. SIMPSON, M. D., F. R. S. E. Prof. of Mid,-wifery in the University of Edinburgh, etc., etc., etc. Philadelphia Lindsay and Blakiston. 1849, 8 vo. pp. 248.

This work is a collection of essays, communicated by Dr. Simpson at different times, for medical journals, together with statements of his experience in the employment of anaesthetic agents, made at meetings of the medical societies of Edinburgh, all of which are now, for the first time, arranged by the author for publication in one volume. The work embraces not only the observations of Dr. Simpson, but results obtained in the different public hospitals of Great Britain, Ireland, and Paris, as well as in the private practice of several eminent practitioners. It contains, also, an epistolatory discussion with Prof Meigs, on the application of anaesthesia to obstetrics.

The name of Dr. S. is identified with the history of anæsthesia, by the introduction of chloroform, as an anæsthetic agent, and the priority of his experience in its employment in obstetrical practice. In the latter investigations he holds the position in Great Britain, which Prof. Channing occupies in this country. Both have rendered valuable service to science by contributing and collecting facts relating to this interesting and important subject: and should future experience show that their enthusiasm has not led them to entertain too sanguine expectations, their names will ever be memorable among those who have conferred signal benefits upon the race.

For sale by G. H. Derby & Co.

